# FDG-PET/CT imaging for staging and target volume delineation in conformal radiotherapy of anal carcinoma

**DOI:** 10.1186/1748-717X-5-10

**Published:** 2010-02-06

**Authors:** Marco Krengli, Maria E Milia, Lucia Turri, Eleonora Mones, Maria C Bassi, Barbara Cannillo, Letizia Deantonio, Gianmauro Sacchetti, Marco Brambilla, Eugenio Inglese

**Affiliations:** 1 Department of Radiotherapy, University Hospital Maggiore della Carità, Novara, Italy; 2 Department of Clinical and Experimental Medicine and Biotechnology Centre for Applied Medical Research, University of Piemonte Orientale, Novara, Italy; 3 Medical Physics, University Hospital Maggiore della Carità, Novara, Italy; 4 Nuclear Medicine, University Hospital Maggiore della Carità, Novara, Italy

## Abstract

**Background:**

FDG-PET/CT imaging has an emerging role in staging and treatment planning of various tumor locations and a number of literature studies show that also the carcinoma of the anal canal may benefit from this diagnostic approach. We analyzed the potential impact of FDG-PET/CT in stage definition and target volume delineation of patients affected by carcinoma of the anal canal and candidates for curative radiotherapy.

**Methods:**

Twenty seven patients with biopsy proven anal carcinoma were enrolled. Pathology was squamous cell carcinoma in 20 cases, cloacogenic carcinoma in 3, adenocarcinoma in 2, and basal cell carcinoma in 2. Simulation was performed by PET/CT imaging with patient in treatment position. Gross Tumor Volume (GTV) and Clinical Target Volume (CTV) were drawn on CT and on PET/CT fused images. PET-GTV and PET-CTV were respectively compared to CT-GTV and CT-CTV by Wilcoxon rank test for paired data.

**Results:**

PET/CT fused images led to change the stage in 5/27 cases (18.5%): 3 cases from N0 to N2 and 2 from M0 to M1 leading to change the treatment intent from curative to palliative in a case.

Based on PET/CT imaging, GTV and CTV contours changed in 15/27 (55.6%) and in 10/27 cases (37.0%) respectively. PET-GTV and PET-CTV resulted significantly smaller than CT-GTV (p = 1.2 × 10^-4^) and CT-CTV (p = 2.9 × 10^-4^). PET/CT-GTV and PET/CT-CTV, that were used for clinical purposes, were significantly greater than CT-GTV (p = 6 × 10^-5^) and CT-CTV (p = 6 × 10^-5^).

**Conclusions:**

FDG-PET/CT has a potential relevant impact in staging and target volume delineation of the carcinoma of the anal canal. Clinical stage variation occurred in 18.5% of cases with change of treatment intent in 3.7%. The GTV and the CTV changed in shape and in size based on PET/CT imaging.

## Background

Carcinoma of the anal canal has shown an increasing incidence over the last decades accounting for approximately 0.5-1 new case per year every 100,000 inhabitants in Western countries [1,2]. The treatment approach moved from an extensive surgical approach consisting of abdominal-perineal resection to a conservative chemo-radiation regimen proposed firstly by Nigro et al. who reported high response and survival rates after a combination of radiotherapy and chemotherapy [3]. The efficacy of such an approach was confirmed by phase III trials [4,5]. Multivariate analysis showed that the two most significant prognostic factors are tumor size and nodal status related to the TNM stage [6]. Consequently, the efficacy of treatment relies on accurate staging of the primary tumor and the regional lymph nodes [7]. Moreover, the precise identification of the radiotherapy treatment volume plays a crucial role in order to avoid geographic miss and appropriately boost nodal disease [8].

(18)F-fluorodeoxyglucose positron emission tomography fused with computed tomography (FDG-PET/CT) imaging has an emerging role in staging and treatment planning of various tumor locations and a number of literature studies show that also the carcinoma of the anal canal may benefit from this diagnostic approach [6,9-13].

The present prospective study aims to analyze the potential impact of FDG-PET/CT in staging and target volume delineation of patients affected by carcinoma of the anal canal and candidates for curative radiotherapy combined with concomitant chemotherapy.

## Methods

### Patients

From January 2005 to May 2008, 27 patients, 9 males and 18 females, aged from 36 to 90 years (mean and median 66 years), performance status of 80-100 (median 90) according to Karnofsky scale with biopsy proven anal carcinoma, were enrolled in the present study after obtaining informed consent following the rules of our institution. Pathology was squamous cell carcinoma in 20 cases, cloacogenic carcinoma in 3, adenocarcinoma in 2, and basal cell carcinoma in 2. Tumor was confined in the anal canal in 10 cases, extended to the lower rectum in 3 cases, to the anal margin in 8 cases and to both these areas in 6 cases. Baseline work-up included physical examination, blood count, renal and liver function tests, endoscopy, CT-scan with contrast of the upper and lower abdomen, and chest X-rays. One patient was HIV positive. The main patient characteristics are reported in Table 1. All cases were discussed in a multidisciplinary conference with surgeons, radiation oncologists, and medical oncologists.

**Table 1:** Main patient characteristics.

Total number	27
Gender	18 females 9 males

Median age	66 (range 36-90)

Median Karnofsky performance status	90 (range 80-100)

Clinical stage (baseline work-up with CT alone)	2 T1 N0 M0
	4 T2 N0 M0
	9 T3 N0 M0
	2 T1 N1 M0
	4 T2 N2 M0
	2 T3 N2 M0
	1 T4 N2 M0
	1 T3 N3 M0
	1 T4 N3 M0
	1 T3 N2 M1

### PET/CT simulation

Simulation was performed by PET/CT hybrid scanner (Biograph 16 HI-REZ, Siemens, Hoffman Estates, IL) with patient in supine position with knee-ankle positioning device to improve the reproducibility at each treatment session. The CT scanner was used both for attenuation correction of PET data and for localization of FDG uptake in PET images. All patients were advised to fast for at least 8 hours prior to PET/CT examination. After injection of 5.18 MBq of FDG per kg of body weight, patients were rested for a period of about 60 minutes in a comfortable chair. Emission images ranging from the proximal femur to the base of the skull were acquired for 3-4 minutes per bed position. Field of view was of 50 cm with a matrix of 512 × 512 pixels for CT and of 128 × 128 for PET. The processed images were displayed in coronal, transverse, and sagittal plans. After image acquisition, PET/CT data sets were sent to the treatment planning system Pinnacle (Philips, ADAC Laboratories, Milpitas, CA) through local network.

### Target volume delineation

**Figure 1:**
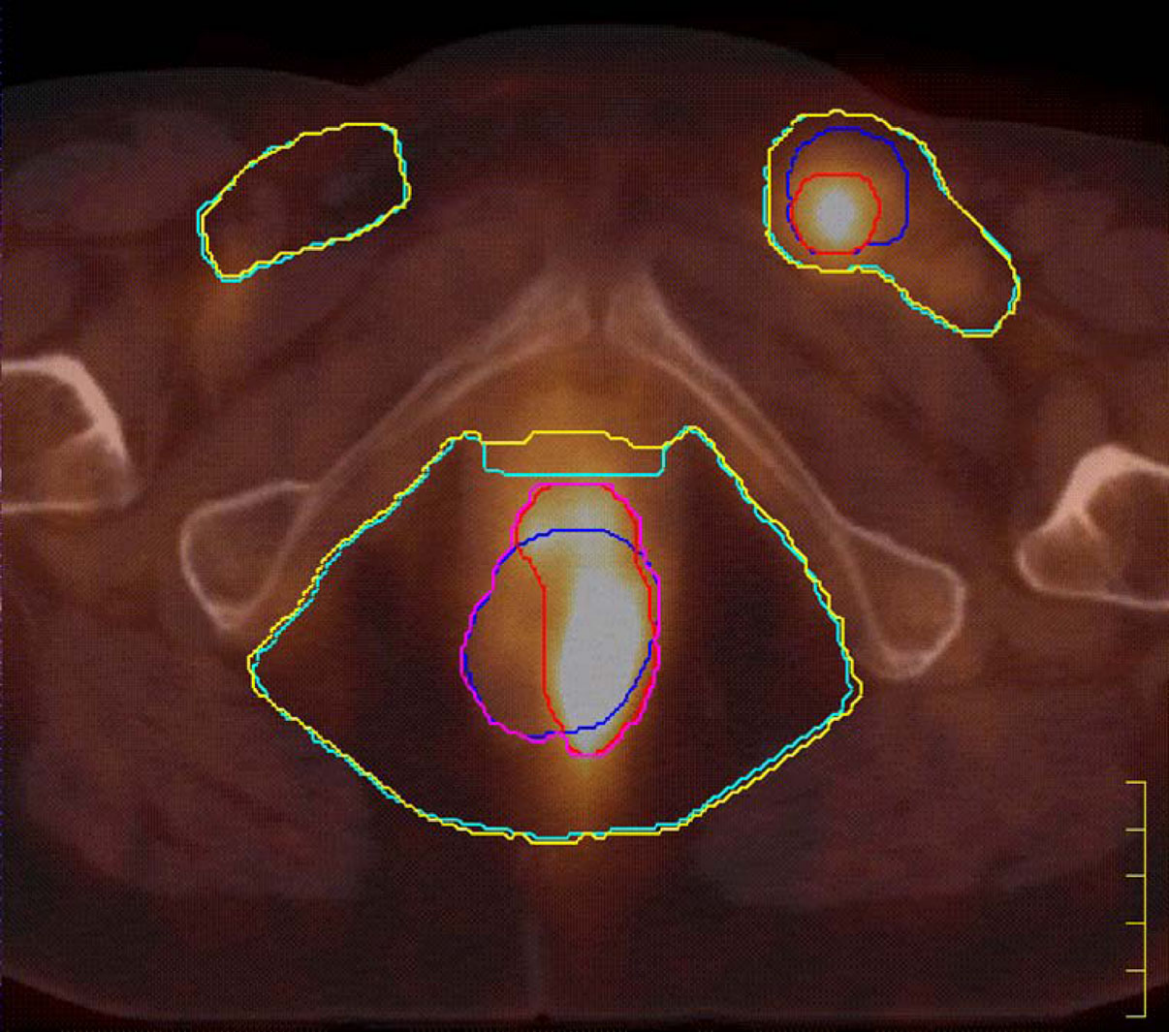
**PET/CT image in axial view of a T3N2 case**. Different colours are used to highlight the contours of the treatment volumes: CT-GTV (blue), PET-GTV (red), PET/CT-GTV (purple), CT-CTV (light blue), and PET/CT-CTV (yellow). The PET/CT GTV and the PET/CT-CTV were used for treatment purposes.

Treatment volumes including GTV, CTV, and organs at risk (bladder and femoral heads) were drawn on CT and then on PET/CT fused images by the same radiation oncologist with a specific experience in the treatment of gastro-intestinal tract tumors. In particular, the GTV was drawn manually on CT and semi-automatically on PET images. For delineating the PET/CT-GTV and the PET/CT-CTV, the operator considered both CT and PET information outlining the volume identified by one or by both imaging modalities. The CTV was drawn manually on CT and on PET/CT images taking into account respectively the CT-GTV and PET/CT-GTV contours. The GTV included the primary tumor extension and the involved lymph nodes. The CTV surrounded the GTV with margins of at least 1.5 cm at the level of the primary tumor and included the uninvolved regional lymph nodal areas, i.e. bilateral inguinal, external and internal iliac, perirectal, and presacral lymph nodes (Figure 1).

Lymph nodes were considered positive at CT-scan when greater than 15 mm in diameter or when containing areas of necrosis. A focus was considered positive at PET when the activity was significantly above the expected background and could not be explained by a normal structure. For delineation on PET/CT images, a fixed threshold value of 40% of the maximum uptake in the lesion (whichever primary tumor, liver metastasis or lymph node) was chosen as described in a previous article on the use of PET/CT in rectal cancer [14]. For treatment purposes, PTV was obtained by 10 mm symmetric expansion of CTV taking into account setup uncertainties and organ motion. The GTV with symmetric expansion of 10 mm was used as a volume for boosting macroscopic disease.

A 3-dimension conformal treatment plan was performed to a total dose of 54.0-65.0 Gy (median 59.4 Gy) to the macroscopic disease and to 45.0 Gy to the potentially microscopically invaded regions with conventional daily fractionation of 1.8-2.0 Gy by using 6-15 MV photons for curative treatments. The case treated with palliative intent was irradiated to a total dose of 35.0 Gy with daily fractionation of 2.5 Gy. Concomitant chemotherapy was given by cisplatin and 5-fluorouracil in 15 cases and mytomicin and 5-fluorouracil in 8 cases.

### Statistical analysis

PET-GTV and PET-CTV were respectively compared to CT-GTV and CT-CTV by Wilcoxon rank test for paired data. A P-value < 0.05 was considered to be statistically significant. Data were reported as mean ± standard deviation and 95% confidence interval (CI) or as experimental percentage with 95% CI, calculated using the binomial distribution.

The following additional volumes were considered for the mismatch analysis:

- the volume identified by PET but not by CT (PEToutCT),

- the volume identified by CT but not by PET (CToutPET),

- the common volume of CT and PET (CT&PET).

## Results

The average tumor-to-background ratio in the examined lesions was 22.0 ± 10.5. Using a 40% fixed isoactivity level of the signal maximum of the tumor, liver lesion or suspicious node, the resulting activity level was always above the background activity.

**Figure 2:**
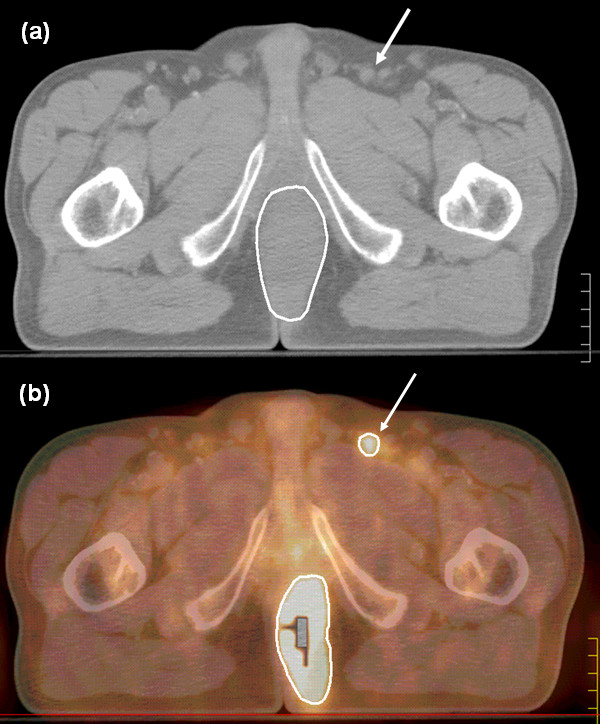
**(a -- b) CT (a) and PET/CT (b) in axial view showing a lymph node of about 1 cm in diameter with intense uptake at PET (arrows)**. Positivity was confirmed by FNAB. Based on these finding, this lymph node was included in the GTV and received a boost of radiation dose.

PET images showed extensive FDG tumor uptake in all but one case of squamous cell carcinoma T3N0 M0 in which the accumulation of the radiotracer was limited to a small portion of the tumor mass detectable by clinical examination and CT. PET/CT fused images led to change the stage in 5/27 cases (18.5%; CI: 6.2% - 37.0%). Three of these changes were related to the lymph node stage that changed from N0 to N2 (Figure 2) and two to the detection of liver lesions showing FDG uptake at PET (Table 2). In the three cases presenting with FDG uptake in inguinal nodes, a fine-needle ago-biopsy (FNAB) confirmed the presence of tumor cells. The detection of liver lesions was confirmed by contrast CT and MRI scans. The case with multiple lesions in the liver was treated with palliative radiotherapy followed by chemotherapy whereas the other case presenting with a single FDG uptake lesion received combined radio-chemotherapy as the other cases treated by curative intent. After radio-chemotherapy, this patients underwent surgical resection of the remaining liver lesion that confirmed the finding of metastasis.

**Table 2:** Change of clinical stage by positron emission tomography/computed tomography PET/CT findings.

Pre-PET TNM	Post-PET TNM	Tumor sites
T3N0 M0	T3N2 M0	Inguinal lymph node *

T3N0 M0	T3N2 M0	Inguinal * and external iliac lymph nodes

T3N0 M0	T3N2 M0	Inguinal *, external iliac, and perirectal lymph nodes

T3N0 M0	T3N0 M1	Multiple liver metastases

T3N3 M0	T3N3 M1	Single liver metastasis

* confirmed by fine-needle ago-biopsy (FNAB)

PET/CT led to change GTV and CTV contours in 15/27 (55.6%; CI: 34.8 - 74.3) and in 10/27 cases (37.0%; CI: 19.0 - 57.6) cases respectively. In particular, changes in GTV contours occurred in 12/15 (80%; CI: 53.0 - 95.7) cases staged T3-T4 and in 3/12 (25%; CI: 5.5 - 56.1) cases staged T1-T2. As far as the volume size, the analysis by Wilcoxon rank test showed PET-GTV and PET-CTV to be significantly smaller than CT-GTV (p = 1.2 × 10^-4^) and CT-CTV (p = 2.9 × 10^-4^) respectively. PET/CT-GTV and PET/CT-CTV, that were used for clinical purposes, were significantly greater than CT-GTV (p = 6 × 10^-5^) and CT-CTV (p = 6 × 10^-5^). The mean difference GTV (9.2 ± 12.8 cc; 95% CI: 4.2 - 14.3 cc) amounted to an average 11.1% of the CT-GTV volume. This difference was in proportion higher than the corresponding mean difference between PET/CT-CTV and CT-CTV (50.3 ± 26.6 cc; 95% CI: 39.7 - 60.8 cc) which amounted, on average, to 5.1% of the CT-CTV volume. The analyzed volumes for all patients are reported in Table 3. The mean and range values of the additional volumes analyzed to compare PET/CT and CT alone are reported in Table 4. In particular, the mean PEToutCT volume was 10.6% of the mean CT-GTV.

**Table 3:** Volumes (cc) identified by CT and PET in every single case.

Patients	CT-GTV	PET-GTV	PET/CT-GTV	CT-CTV	PET/CT-CTV
1	118.1	31.3	120.1	1003.8	1044.4
2	102.4	62.2	121.8	1154.4	1226.6
3	117.6	54.3	128.8	827.3	872.1
4	46.0	33.6	46.2	852.0	889.8
5	358.4	312.1	373.2	1334.0	1370.9
6	45.1	29.1	48.2	907.5	938.1
7	88.9	53.0	90.7	1266.2	1277.7
8	57.9	24.4	74.3	1077.9	1138.8
9	107.4	101.5	141.5	1038.7	1066.6
10	72.9	27.5	73.9	792.6	815.7
11	129.8	27.7	132.4	998.1	1034.2
12	31.7	9.6	32.1	685.3	711.4
13	24.5	17.8	31.3	1007.6	1046.8
14	161.3	175.8	218.2	1034.3	1146.3
15	206.1	84.5	222.1	1040.3	1121.5
16	56.8	27.8	68.9	859.3	910.9
17	82.4	21.3	82.4	812.2	847.4
18	56.7	16.9	59.9	1003.4	1040.8
19	86.6	81.7	108.2	1310.1	1380.5
20	38.4	36.1	47.9	996.5	1100.1
21	75.7	32.0	76.1	1119.1	1152.8
22	85.6	38.3	93.9	958.6	1044.2
23	42.8	11.9	43.4	1266.6	1335.6
24	27.7	12.1	28.2	854.7	919.2
25	74.0	46.9	76.5	960.8	977.1
26	13.6	13.3	17.1	734.1	760.2
27	28.0	7.1	28.1	883.8	966.1

Mean ± SD	86.5 ± 70.2	51.5 ± 63.2	95.7 ± 76.2	991.8 ± 171.2	1042.1 ± 178.1

**Table 4:** Volumes (cc) identified after fusion of PET and CT images.

Volumes	Mean	Range	Confidence Interval
PET/CT-GTV	95.7	17.1-373.2	65.5 - 125.8

PEToutCT	9.2	0.0-56.9	4.3 - 14.1

CToutPET	44.3	3.8-137.5	32.2 - 56.4

CT&PET	42.3	6.9-297.3	20.0 - 64.6

After median follow-up of 18 months (range 3 - 42 months), loco-regional control was obtained in 18/27 (66.7%) cases and disease-free and overall survival (DFS and OS) rates were 66.7% and 77.8% respectively. Acute and late toxicity higher than grade 2 (RTOG/EORTC) was observed in 7/27 (25.9%) and 1/27 (3.7%) patients respectively.

## Discussion

The present study, like others reported in the literature, analyzed the potential impact of PET/CT images on tumor staging and treatment strategy and is one of the first reports examining quantitatively how the GTV and the CTV for radiotherapy treatment planning may change in relation with the use of functional imaging.

As a matter of fact, a number of recent literature studies tried to show that the addiction of PET and PET/CT may be able to add useful information for the carcinoma of the anal canal. Most of theses studies focused on disease staging with special regard to nodal spread whereas relatively few of them tried to analyze the impact of PET/CT on radiotherapy treatment plans. Trautmann et al. reported that pre-treatment PET, in a series of 21 patients candidates for radiotherapy and chemotherapy, changed disease staging in 24% of cases in relation to lymph nodal, omental, and liver metastases [9]. Cotter et al. analyzing 41 patients affected by anal carcinoma, observed that FDG-PET/CT detected abnormal nodes in 20% of groins with normal CT appearing leading to upstage 25% of patients [10]. Conversely 23% of CT-positive lymph nodes were PET negative. Notably, PET was positive in 17% of cases negative both at CT and physical examination. More recently, Nguyen et al. found that PET upstaged 17% of patients with unsuspected pelvic/inguinal nodal disease [6]. Another study on 61 patients by Winton et al. reported a change in tumor stage in 23% of cases as a result of PET/CT imaging with 15% of upstaging and 8% of downstaging [13]. The same authors observed a change in treatment strategy in 3% of patients.

In the present series, we observed similar data with change in staging of 18.5% and in treatment strategy of 3.7% of cases. As reported in other studies, most changes were found at the level of the lymph nodes, typically in the inguinal regions even with node diameter < 1.5 cm (Figure 1). In the present study, we did not observe any downstaging of the disease possibly related to case selection of our series. As far as the primary lesion, a case of squamous cell carcinoma showed FDG uptake only in a part of the tumor mass detected by CT and clinical examination. This finding could be related to a relatively low metabolism of the tumor cells of the PET-negative component of the lesion.

The impact of PET/CT imaging on treatment planning was analyzed only by few authors without specific quantitative analysis of the treatment volumes. Anderson et al. in a series of 20 anorectal tumors found that PTV changed, based on PET data, in 1/3 patients and Nguyen et al. observed a significant change of the treatment plan in 19% of cases because the upstage of regional lymph nodes [6,11]. More recently, Winton et al. reported a modification of radiotherapy fields in 13% of patients after PET/CT imaging [13].

In our series, the GTVs and the CTVs detected by CT alone and by PET/CT images were analyzed in details and compared. The contours outlined on PET/CT imaging were substantially different from those outlined on CT alone, more frequently for the GTV (55.6%) than for the CTV (37.0%). This findings may be related to the different shape of GTV detected by the two diagnostic modalities and strongly influenced by PET uptake and to the relatively constant shape of CTV, i.e. the potential microscopic tumor extension, typically defined by regional anatomical landmarks on CT imaging and less influenced by PET imaging. These changes were more evident for the T3-T4 cases (80% of changes) where CT images may be unable to clearly detect the tumor extension in relation with the close proximity of muscle structures especially at the level of the perineum. As a matter of fact, CT images may overestimate tumor volume in low rectal cancer as observed by O'Neill et al. who compared MR versus CT imaging [15].

PET/CT imaging influenced also the size of the treatment volumes: more that of the GTV than that of the CTV. In particular, the GTV defined by FDG-PET uptake (PET-GTV) was significantly smaller than the GTV detected by CT alone (CT-GTV) despite a number of lymph nodes detected only by FDG-PET. This difference in size may be related to at least two different factors: on one side, CT images may include not only the metabolically active tumor but probably also other tumor components and possibly tissue alterations surrounding the tumor itself; on the other side, PET images may be able to highlight the metabolically active aspect of the tumor but not the necrotic or slow growing tumor components. The volume used for treatment purposes, i.e. the PET/CT-GTV, was significantly greater than the CT-GTV meaning that PET uptake may extend also beyond the tumor volume visible on CT images. This information could lead to reduce the risk of geographic miss at the periphery of the tumor. Similarly, the PET/CT-CTV was larger than the CT-CTV in relation to the margin around the tumor extension and the additional lymph nodes detected by PET. This difference was evident but less pronounced than that between PET/CT-GTV and CT-GTV because the CTV is related to the concept of treating the microscopic disease undetectable by imaging modalities.

The present study has some limitations. One is represented by the reliability of PET uptake in detecting tumor tissue considering that data about sensitivity in particular at the level of the regional lymph nodes is quite high (67-100%) but the specificity cannot be ascertain because of the lack of complete pathology surgical data correlated with PET findings [13,16]. In our experience, we performed FNAB at the level of the inguinal nodes but we did not have pathology data on the other tumor components. Another issue is related to the interpretation of PET uptake in terms of threshold value to determine the real size of a positive lesion. In this regard, we adopted the fixed threshold of 40% suggested also by other authors and already used in a previous study about rectal cancer, though the determination of the most appropriate method for contouring PET images is still under investigation [14,17-19]. However, the very high average tumor-to-background ratio measured in our sample renders unlikely a significant change in PET volumes using adaptive threshold algorithms, which usually reach a plateau of about 40% in this range of tumor-to-background.

Data in terms of loco-regional control and toxicity do not substantially differ from others reported in the literature but follow-up time is too short to make any consideration about DFS and OS [2,5,6,13].

## Conclusions

The present study showed that FDG-PET/CT imaging has a potential relevant impact in staging and target volume delineation of the carcinoma of the anal canal. Clinical stage variation was observed in 18.5% of cases with change of treatment intent in 3.7%. GTV and CTV contours changed in 55.6% and 37.0% of cases respectively. PET/CT-GTV and PET/CT-CTV, that were used for clinical purposes, were significantly greater than CT-GTV and CT-CTV. These variations in treatment volumes may become very relevant when using highly conformal techniques like intensity modulated radiation therapy or particle therapy.

## Abbreviations

FDG-PET/CT: (18)F-fluorodeoxyglucose positron emission tomography fused with computed tomography; HIV: Human Immunodeficiency Virus; MBq: Mega Becquerel; GTV: Gross Tumor Volume; CTV: Clinical Target Volume; PTV: Planning Target Volume; CI: Confidence Interval; FNAB: Fine Needle Ago Biopsy; SUV: Standard Uptake Value.

## Competing interests

The authors declare that they have no competing interests.

## Authors' contributions

MK was the study coordinator, participated in the development of the study and drafted the manuscript. MEM, LT, CB and LD were involved in data collection and review. EM, BC and MC worked on analysis of data. EI and MB participated in the design of the study and contributed to write the manuscript. All authors read and approved the final manuscript.
